# Implementation of case studies in undergraduate didactic nursing courses: a qualitative study

**DOI:** 10.1186/1472-6955-12-15

**Published:** 2013-07-04

**Authors:** Danette K Dutra

**Affiliations:** 1 College of Health and Human Services, Department of Nursing, California State University, Fresno, 2345 East San Ramon Avenue M/S MH25, Fresno, CA 93740-8031, USA

**Keywords:** Case studies, Critical thinking, Facilitative learning, Learner centered, Nurse educators, Pedagogy, Scenario-based case studies, Unfolding case studies

## Abstract

**Background:**

The implementation of unfolding scenario-based case studies in the didactic classroom is associated with learner-centered education. The utilization of learner-centered pedagogies, such as case studies, removes the focus from the instructor and instead places it on the student. Learner-centered pedagogies are believed to improve students’ levels of cognition. The purpose of this study was to examine how nurse educators are implementing the pedagogies of case studies in their undergraduate didactic courses. The goal was to examine, document, report, and, ultimately, implement the strategies.

**Methods:**

Purposeful sampling was utilized in this qualitative, multisite-designed study. For each of the four participants, three separate site visits were completed. Observations and post-observational interviews took place at each site visit. Transcribed data from interviews, observations, and course documents were imported into the computer program *Nvivo8*. Repetitive comparative analysis was utilized to complete the data coding process.

**Results:**

The guiding research question of this study sought to investigate the implementation strategies of case studies in didactic nursing courses. The implementation of case studies by the participants reflected two primary patterns: Formal Implementation (FI) and Informal Implementation (II) of case studies. The FI of case studies was further divided into two subcategories: Formal Implementation of case studies used Inside the Classroom setting (FIIC) and Formal Implementation of cases studies used Outside of the Classroom (FIOC).

**Conclusion:**

Results of this investigation have led to an increased understanding of implementation strategies of unfolding scenario-based case studies in undergraduate nursing didactic courses. Data collected were rich in the description of specific methodologies for utilization of case studies and may serve as a resource for faculty in development of creative strategies to enhance the didactic classroom experience.

## Background

The level of competency at which a graduate from nursing school must perform has been raised. This escalation of the competency level for nurses has been attributed to the increase in the complexity of patient conditions and the increase in technological skill required to practice in a complex healthcare environment [1-6].

Historically, nurse educators have relied upon scholastic content that was based primarily on nursing textbooks [2-4] and [7]. Students listened to lectures for extended periods of time, often for 3 hours straight, with only occasional breaks. This approach was very instructor-centered. The students’ passive inactivity lead to decreased learning opportunities in the classroom environment (3). The concern now is not that the old pedagogies once relied upon are no longer sound techniques, but that nurse educators have not adapted their pedagogies to include newer methodologies that are believed to enhance their students’ levels of thinking and reflect practices in the current healthcare setting [1,3,4].

Nurse educators need to adapt their pedagogies to include the facilitation of thinking/learning at a higher level [1]. Learner-centered classroom environments promote learning that is at the analysis, synthesis, and evaluation level of Bloom’s taxonomy of cognitive educational outcomes. This level is indicative of a higher level of cognition and is often associated with long-term recall [8]. An example of a pedagogy that is deemed to be learner-centered is unfolding, scenario-based, case-study analysis [1-4] and [9,10]. The purpose of this study was to examine how nurse educators are implementing the pedagogies of case studies in their undergraduate didactic courses. The anecdotal documentation of pedagogical implementation of case studies, by exemplar educators, provides a foundation for future investigation of effective teaching strategies, potentially leading to evidence-based practice guidelines for nurse educators.

### Educational significance

Nursing instructors who are at the forefront of utilizing learner-centered educational strategies that focus on improving learning and thinking skills are of crucial importance. Without the knowledge of how these effective pedagogical components are implemented, the quest to disseminate effective teaching strategies to other nurse educators is at risk of being stifled. The goal of this study was to uncover the pedagogical methods being employed by those nurse educators who have achieved a learner-centered classroom environment. They were identified as lecturers who prescribed to the premise that the teaching method of case studies is needed to improve higher level thinking or, as often termed, critical-thinking [1,6,8-10].

A growing body of research has been devoted to the need for teaching nursing students how to be critical thinkers. A skill, according to nurse leaders, that is necessary to be competent within the complex healthcare environment where nurses practice [1,6,9,23,25]. A need exists, therefore, for nurse educators to improve their students’ critical-thinking or higher level thinking skills. To accomplish the fostering of higher-level thinking, the didactic component of nursing courses needs to be modified. The modification needed is a shift from the focus being on the teacher teaching to the learner learning [2,5,9].

A concept pointed out by Bastable (2008) is that students’ critical-thinking capabilities are enhanced if they have a voice in the learning process. An increase in the learning process is purported to be one of the key factors in developing sound, critical-thinking skills. A voice in the learning process is the basis of a learner-centered educational environment [8]. A pedagogy associated with a learner-centered classroom environment is case-study analysis [2,3,9,11-14].

### Conceptual framework

To establish an understanding of how effective nurse educators are implementing the teaching strategy of case studies, this study utilized two theoretical constructs: Information Processing Theory (IPT) [16] and Dimensions of Thinking Framework (DTF), established by Marzano et al. [15]. Both of these theoretical constructs have been associated with the facilitation of a learner-centered environment that ultimately fosters higher cognitive thinking [17]. Both constructs were the lens by which this study attempted to conceptualize the teaching pedagogies of participants in this study. The two conceptual frameworks were not meant to be used as an evaluation tool of participants’ pedagogies; instead, they were employed as a method to describe the implementation strategies utilized by effective educators and the reasoning behind their utilization.

#### Information processing theory

The IPT is a collection of concepts that has its roots within cognitive psychology. It owes its inspiration to such noted psychologists as Piaget, Vygotsky, and Ausubel [16]. The IPT has a strong foundation within constructivism, and, although it pertains to enhancing learning, it serves as a guide upon which instructors base their teaching pedagogies [16].

The IPT contains six key components, each of which acts as a framework in which to view the specific teaching methods employed by the studies participants.

*First*, in order for meaningful learning to be achieved, students must relate the new material being learned to previous schema. Nurse educators who deliberately link knowledge learned in previous courses with current course material have chosen to adhere to the concept of linking schemas [8].

*Second and third*, a new concept being presented must be organized in its delivery and presented at the appropriate education level for the students [8].

*Fourth*, students can handle only a given amount of new material at a time. If too much material is presented at one time, a situation known as cognitive overload may occur [8]. Often, didactic nursing courses are 3 hours long. Given the comprehensiveness of the material covered in most nursing classes and the length of time students are in class, cognitive overload is all but guaranteed without some sort of varied teaching approach. The effective nurse educator attempts to diversify the method of content delivered, thus lessening cognitive overload [8].

*Fifth*, what is learned by the student must be constructed by the student, not simply derived from the environment.

*Sixth*, students need to be active in the learning processes. The final two components are considered necessary to the enhancement of students’ awareness of how they learn, which in turn improves their learning capabilities [16,18].

Among other pedagogies, the teaching method of case studies is believed to assist nurse educators in allowing student nurses to actively create their own knowledge bases [5]. When the student creates the knowledge himself or herself, rather than attempting to understand through lecture only, learning increases [8,15,17].

The use of case studies as a means by which to present a lesson ultimately allows students to make their own decisions regarding plans of care for patients. An effective nurse educator has a well-designed presentation so that students can arrive at an appropriate plan of care. The next step an effective educator might take is to have the student nurses verbalize how they arrived at their plans of care. Thus, students acknowledge their own leaning processes [5].

#### *Dimensions of thinking framework*

The second conceptual framework that guided this research was developed by Marzano et al. (1988): *The Dimensions of Thinking Framework*. According to Marzano et al., the identification of key dimensions of thinking was needed to provide educators with a framework to teach thinking. This framework focuses on four dimensions of thinking. Although Marzano labeled the dimensions as separate entities, he cautioned they were not designed to offer a hierarchy and actually represent a thinking continuum. Each dimension is seen to overlap and complement one another [15,17]. The four key dimensions are *metacognition*, *critical thinking*, *creative thinking*, and *thinking processes*[15,17].

*Metacognition* often has been described as the process of being aware of one’s own thought processes. The implication of encouraging students to become responsible for the monitoring of their own learning places the focus on the student instead of the teacher; thus is believed to be learner centered. The transfer of responsibility in and of itself constitutes a higher level of cognition. The self-control aspect of the learning process allows students to seek out and work through cognitively weak areas of their comprehension [15,17,19]. The ability of students to evaluate what and how they think establishes a means by which they become lifelong learners, which all nurses ultimately must accept as their destiny [1,3].

According to Marzano, key pedagogies that an instructor should implement to enhance metacognitive skills include deliberate planning of activities that are designed to make the students question, analyze, and evaluate a given concept or process [15,17]. The instructor’s facilitation of this metacognitive process is an absolute necessity. However, the student is the center of the process.

*Critical* and *creative thinking* are seen as complementary. They do not represent two divergent thought processes. For example, pedagogies designed to enhance students’ critical- and creative thinking skills need to include exercises that have more than one right answer. Given the fact that the environment that nurses practice in can change instantaneously, the need for instructors to have students practice thinking about diverse points of view in the didactic lecture environment enhances the transfer of learning by student-nurse to real-life nursing practice [3,10].

Within the DTF, the idea of “Thinking Processes” is seen to include simple cognitive skills, such as comprehension of specific principles and further includes the attainment of more complex cognitive ability, such as researching, composing, and problem solving. When designing curriculum to include all of the thinking processes, the instructor should first establish the key concepts and principles that need to be learned by the students. Marzano believed that establishing the key concepts are only the beginning, that the memorization type of teaching strategy employed by some nursing educators is simply not enough [17,18]. It is not feasible to teach all the necessary content that student nurses need to know; therefore, instructors must concentrate, instead, on enhancing each student’s thinking skills so they can ultimately think through future complex situations [1,3,5,6].

This ideology explicitly pertains to the education of student nurses. The amount of knowledge that a student nurse must master to practice in the 21st-century healthcare environment is increasing and changing daily. Therefore, according to Ironside, instructors need to stop adding content to an already packed curriculum and instead teach student nurses how to think beyond what content there is time to present [2].

Effective nurse educators who realize the importance of how information is processed and have an understanding of the dynamic learning phases are a step ahead of other nurse educators when it comes to implementing teaching strategies that lead to higher levels of cognition. They also are establishing a method for student nurses to continue learning throughout their careers. The use of the IPT and the DTF allowed for the identification of the reasoning behind the methodologies employed by participants in this study. Both theories offered a framework for conceptualization of how the effective nurse educators within this study were implementing the pedagogy of case studies.

Research questions

1. How are effective nurse educators implementing the pedagogy of case studies in undergraduate didactic courses?

2. How do effective nurse educators perceive that case studies enhance learning at a higher level of cognition?

## Methods

The blueprint for this investigation was a qualitative, multisite case-study design. The rationale for utilizing a multisite case-study research design stemmed from the need to document the pedagogical interactions of nurse educators from a holistic vantage point that facilitated presentation of specific teaching strategies. Viewing the educators holistically, in their own environments, allowed increased insight into their methodologies of case study implementation. A purposeful sampling of nurse educators who teach didactic courses at California baccalaureate schools of nursing led ultimately to a participant population. Inclusion in the study was based upon two criteria: (a) the participant had been referred by an individual who was a current administrator within a California baccalaureate school of nursing (public and private institutions) (It was assumed that participant were utilizing case studies in their didactic courses.) and (b) an inventory score on the Orientations to Teaching Questionnaire (OTQ) identified the participant as primarily learner-centered in his or her approach to teaching in a didactic educational setting. The OTQ is a tool that was developed by Kember and Gow and identifies the instructor’s orientation or approach to didactic education at the college level [21]. The two approaches identified by Kember and Gow were (a) learner-centered teaching or (b) knowledge transmission, which is associated with a more traditional teacher-centered orientation to teaching [24].

A sample size of four faculty ultimately was achieved. Written consent was obtained from each of the participants. Participation was assured to be on a volunteer basis and each participant was advised that she could have resigned from the study at any time during the process. To ensure confidentiality, the participants were identified with both pseudonyms for themselves and for the schools of nursing they were associated with. Their pseudonyms were Amy, Betty, Carol, and Dana (see Table 1).

**Table 1:** Demographics and pseudonyms assigned

**Pseudonym**	**University**	**Location of University**	**Years as nurse**	**Highest level of education**	**Years teaching**	**Type of course teaching**	**Semesters teaching course**
Amy	B/Public	Central	20-25	PhD	5-10	Maternity research	4
							2
Betty	C/Private	Southern	5-10	EdD(c)	< 5	Medical/surgical	> 4
Carol	A/Private	Northern	> 26	Post MSN	5-10	Pharmacology	> 4
						Pathophysiology medical/surgical	> 4
							> 4
Dana	D/Public	Northern	> 26	Post MSN	> 26	Physical/assessment pathophysiology	> 4
							> 4

*Note*: *EdD (c)* Doctorate in Education advanced to candidacy.

Collection of data was fourfold. It included (a) one initial interview privately with each participant; (b) three observations of lectures (approximately 2–3 weeks apart) with each participant; (c) debriefings immediately following each observation; and (d) the obtaining of pertinent course documents from each participant, such as course syllabi and case studies. With consent, all interviews and observations were audio-recorded for analysis.

Bogdan and Biklen compared a case-study design with that of a funnel [20]. At the top of the funnel, at its broadest point, case-study research maintains a holistic view. Data are collected by observing, interviewing, and reviewing pertinent documents obtained from each case. As the funnel begins to narrow in width, so does the next step in a case-study design; thus, the tapering of collected data is initiated. This narrowing of the data is accomplished via coding.

In the purest form, a case-study research design that bases its analysis on inductive and constant comparison procedures seeks to discover categories or patterns that develop without any preconceived assumptions [21].

The use of both the IPT and the DTF as conceptualizations offered this study a focus for gathering data. Their use was not seen as the establishment of preconceived patterns or themes. Their ideologies were utilized as a supportive framework for data collection rather than a tool for compartmentalization of data. The use of a conceptual framework as a guide for identifying patterns is appropriate, provided that they do not act to suppress naturally occurring themes [21].

Case-study qualitative research is based on the ideology of a postpositivism viewpoint. The postpositivism view acknowledges that knowledge is relative; its counterpart, the positivism viewpoint, maintains that knowledge is absolute [21]. Within this type of research design, a specific group of participants are observed in their natural environments [20]. This study observed in an unobtrusive manner the naturally occurring events that took place in California baccalaureate schools of nursing while effective nurse educators taught their didactic nursing courses. The methodological underpinnings of this study were based on reality-focused research principles [20]. Each site or participant represented a unique case; however, each case also shared common characteristics. This researcher’s rationale for utilizing a multisite, case-study research design stemmed from the need to document the pedagogical interactions of effective nurse educators from a holistic vantage point, a view that eventually allowed a detailed description to be presented.

### Data analysis

Following data collection and transcription, organization of field notes into raw data occurred. Open coding was then performed. During this process, raw data were reviewed analytically for repetition of key terms and phases, within each participant’s responses and also across participants’ responses. Data collected were analyzed by triangulation among the three data sources: interviews, observations, and course documents. Results reflected coded data that had been collapsed into patterns. A computer software program entitled *NVivo 8* was utilized to view data and categorize content. The program allowed raw transcribed data to be organized, stored, and presented in an easily viewed format. The terms that were coded were viewed by the lens of the two theoretical frameworks, ultimately leading to themes that directly answered the two research questions.

As an external verification, a peer-review process was performed. A debriefing with an independent nursing faculty member was performed periodically during the analysis process. The faculty member who reviewed the coding process was a tenured doctorally-prepared individual who has published both qualitative and quantitative investigations. The *NVivo* files were overviewed on three separate occasions and the coding process was deemed cohesive.

### Ethical consideration

In accordance with the Institutional Review Board (IRB) at the University of San Francisco (USF), San Francisco, California – United States of America and the American Psychological Association’s ethical principles, participants were informed in written form and provided with verbal clarification, as needed, of the study’s details. Approval from the Human Subjects IRB at USF was received. The identification number for approval is #11047343.

## Results

Research question 1

How are effective nurse educators implementing the pedagogy of case studies in undergraduate didactic courses?

The implementation strategies of case studies by the faculty yielded two primary patterns: (a) Formal Implementation (FI), and (b) Informal Implementation (II) of case studies. The FI of case studies produced two subcategories: (a) the use of Formal Implementation *Inside the Classroom* (FIIC) and (b) the use of Formal Implementation of case studies *Outside the Classroom* (FIOC). The pattern of FI case studies indicates that the case studies were preplanned and were in written format. The students had access to the cases before class or were given them during class either via a PowerPoint® slide or on a separate sheet of paper. The pattern II of case studies is meant to reflect the impromptu use of case studies that were delivered verbally during class time. They were not written down in any form. These informal case studies were often a reflection of personal clinical experiences of the nurse educators. See Table 2 for culminating themes, and Tables 3 and 4 for examples of emerging patterns that ultimately lead to designated themes for both FI and II case study presentations. See Table 5 for a comparison of the two guiding theoretical frameworks and the developed themes for research question two.

**Table 2:** Patterns of implementation of case studies in didactic nursing courses

**Pedagogy**	**Patterns**
Case studies	1. Formal Implementation of Case Studies (FI)
	1a. Inside the Classroom (FIIC)
	1b. Outside of the Classroom (FIOC)
	2. Informal Implementation of Case Studies (II)

**Table 3:** Patterns and subcategories of formal implementation of case studies in didactic nursing courses

**Pedagogy**	**Patterns**
Formal Implementation (FI)	Preplanned written format with due dates some completed as an individual project some as a group
Formal Implementation Inside the Classroom (FIIC)	Case Presentations
	Prearranged Classroom presentations
	Group and Individual
	Role Playing (Impromptu & Structured)
	Student to Faculty
	Student to Student
	Case Study Day
	No prior information – Group Processes
	Problem based learning with structure
	Faculty as facilitator
Formal Implementation Outside the Classroom (FIOC)	Assigned Case Studies with due dates
	Group and Individual projects
	Summative and Formative evaluation
	Discussion Board Case Studies
	Monitored by Faculty
	Template specific case study reviews
	Concept Analysis
	Student chooses patient from clinical
	Structured assignment format with due dates

**Table 4:** Patterns of informal implementation of case studies in didactic nursing courses

**Pedagogy**	**Patterns**
Informal Implementation (II)	The sharing of personal clinical experiences in an unfolding case study presentation
	Not noted in syllabus or power points
	Impromptu
	Mini case studies
	Anecdotal notes about nurses experiences
	Frequently implemented throughout lecture
	Link patient symptoms to nursing care
	Emphasis on emotional aspect
	Structural
	Repeated use of impromptu cases (planned)

**Table 5:** **Research question 2**: **themes presented by conceptual framework**

**Theme**	**Information processing theory**	**Dimensions of thinking framework**	**Participant comments**
Students active	Learner centered	Metacognitive	“My classes are interactive”
			“Engage them with active learning”
			“Students think instead of just listen”
			“Stimulate their critical thinking”
			“Come to their own realizations”
Students integrating	Knowledge constructed by student	Critical thinking	“Visually connects content to care”
			“Makes care come alive”
			“Links lecture to patient care”
			“Practice thinking their thinking”

### Examples of formal implementation of case studies inside the classroom

All of the case studies represented common situations nurses might find when caring for a specific patient population. Amy utilized cases within the classroom by presenting a situation and then having the students carry out the case with impromptu role playing. For instance, one of her cases involved the students interviewing an expectant mother regarding illicit drug use before and during her pregnancy. Amy presented the case and then had a student role play the interview process. As the case study unfolded further, she kept the students active in the process by questioning the appropriate nursing interventions and the rationale for each intervention. Betty and Dana used an even more formal methodology. Students brought case studies into class and then, as a group, responded to prearranged questions. Dana sometimes had the students break into small groups to work through a case; she then had the class come back together as a whole and each group discussed their responses.

Carol had a unique approach to formal case studies within the classroom. Within Carol’s syllabus, the session was represented by “case-study day.” Students were told to bring their reference books and laptops to class. No other information was provided. The class started with Carol having her students break-up into groups. She handed out a case study to each of the student groups. She advised the students there would be no lecture, only a series of case studies that the students were going to analyze and present to the class. Carol termed this case study approach a form of problem-based learning.

One example of a case involved a Hispanic male who did not speak English, lived 70 miles away from the nearest hospital, and had to depend on family members for transportation. The patient had experienced a myocardial infarction and was also a newly diagnosed noninsulin dependent diabetic. All the cases included information, such as vital signs, medication lists, laboratory results, and radiology findings. Each group had a different case to present. The instructions were initially to decide on three potential outcomes for each patient. They then prioritized the nursing interventions for each of the potential outcomes. Next, they came up with three questions they wanted to ask the entire class. Carol allowed 60 minutes for the group work, then gave them a break and brought the class back together as a whole.

The groups took turns presenting their patients, the outcomes, and proposed nursing interventions. They led discussions regarding the three questions they asked the class, which were a mixture of thought-provoking questions and factual questions specific to nursing care. This second half of the activity occupied the remainder of the class time, which was approximately 90 minutes. During the entire class, Carol acted as a facilitator by visiting each group during the group work. Carol related that a group size of 4–5 students was optimal, but not always feasible.

### Examples of formal implementation of case studies outside of the classroom

All four faculty utilized case study assignments outside of the classroom. Some of the case studies were assigned with grades given, and some were formative. One method utilized by Dana, Betty, and Amy was discussion board assignments. Case studies were posted on the course management system, Blackboard®. The discussion board responses from the students were monitored for participation points by the instructor. Carol utilized outside of the classroom assignments in a slightly different manner. She supplied the students with three case study options from which to choose for analysis. Students took the case studies home and worked on them. She gave each student a template of how the case should be presented. Carol encouraged study groups to work on the case studies at home, but required individual submission of case analysis.

Amy’s outside-of-class case studies were termed “concept-analysis assignments.” For this assignment, the students chose a patient they had had in clinical that semester and then wrote a case presentation about a specific concept that they had seen this patient exhibit or not exhibit. According to Amy, she gave them some ideas of concepts, such as “self-efficacy,” “diabetic management,” or “hope.” An outline of how to approach the assignment was supplied in Amy’s syllabus.

### Examples of informal implementation of case studies

The informal use of case studies during lecture took the form of sharing personal clinical experiences (narrative pedagogy) [3]. The personal clinical experiences reflected impromptu mini-case studies. Each of the four participants responded that they used personal experiences liberally within their lectures to present specific concepts. Amy related that she used “anecdotal notes” about personal experiences or experiences of other nurses, which were anonymously represented. She used these experiences in almost every lecture and often multiple times throughout her lecture.

Betty stated, “It just happens. I’m an oncology nurse and I have lots of stories.” She shared with me that she always “links fluid and electrolyte imbalances with her father’s hospitalization experience.” Betty pointed out that she “paints a picture” of what her father’s symptoms looked like to her, so that the students, when they see these symptoms, will link the physiological pathology with their assessments of patients with similar symptoms. Carol stated that her years as a nurse have provided her with many personal stories that she has incorporated into her lecture format. She further explained that it is not only the physiological aspects of her mini-cases that she included in her stories but also “the emotional components of them as well.” Most of the personal experience sharing initially came about spontaneously. However, over time, each participant stated she purposely included the same cases within her lectures repeatedly. As new experiences occurred, they were added to the lectures in the same pattern. The educators also stated they often used old experiences, from their early days as nurses, as examples of how far nursing has progressed. According to Dana, the sharing of real-life examples provided examples of how nursing care constantly changes; she used her old nursing stories to stress the importance of staying current with academic nursing journals and evidence-based practice.

Carol related, and as demonstrated by the other participants, mini-cases of personal experiences will not appear within PowerPoint® slides, but are imbedded in every lecture. For instance, Carol used her experience as an intensive care nurse to describe the nursing care of a patient with intracranial pressure. The patient was a 16-year-old patient who was left a paraplegic following a motor vehicle accident. She was discussing the purpose of intracranial monitoring and related a specific incident regarding utilizing the monometer at the appropriate height.

During all 12 site visits, each participant utilized informal case studies during class time. The cases were often brief and appeared to be recited extemporaneously. Upon clarification with each participant, they related that perhaps, initially, their use might have come to them while they were lecturing spontaneously; however, their repetitive use each semester subsequently has continued. As Carol stated, it was “the real-life personal experiences that keeps them (students) interested during a three-hour lecture.” According to the Amy, the students want to know about “real-life” nursing.

Research question 2

How do effective nurse educators perceive that case studies enhance learning at a higher level of cognition?

Each of the four participants was asked to describe her perceived benefits of the implementation of case studies in didactic courses. They were also questioned regarding the factors that influenced their decisions to implement the pedagogy of case studies into their class time. According to the participants, the students were asked to participate in the learning process when they used case studies. They have to “think,” according to Dana. She believed that the use of case studies has helped integrate the content within the didactic portion of nursing school and the clinical. Betty stated that case studies are “nursing in action.” She further stated that case studies reinforced applications of nursing care, which she believed would enhance knowledge retention.

Carol had a term for instructors who adhered to a traditional lecture format in their didactic course and never varied their pedagogies: *the talking head*. She explained that she had been trying to be more of a guide to learning in the classroom rather than the person simply standing at the front of the room doing all the talking. Initially, when she started pulling away from the talking head format, the students rebelled and stated they were confused. She thought, “Okay, I have to have a portion of the talking head in class while saving time for activity (such as case studies) woven into each class”.

Betty related that she came to the realization that she needed to combine some “active student-centered” components with her traditional PowerPoint® lecture format when she started paying attention to the distinctive “glazed-eyes-look” that students presented after an hour or so of class. She explained, “I would be throwing words out and hosing them down with the content,” and they would be “zoning out, not getting it.” Betty concurred with Carol that you cannot eliminate completely the traditional lecture format because students have a certain comfort level with this tradition; however, both are quick to defend the addition of an active-learning environment.

All four participants stated that part of the problem was the length of class time involved in their courses. All of them had class times that were 3 hours long, once a week. To keep the students active in the learning process, they stated they combined a learner-centered pedagogy and lecture with traditional PowerPoint® presentations on a routine basis. Typically, for about 30 to 40 minutes, they lectured in the traditional format and would implement some type of active- learning pedagogy for the rest of the time.

## Conclusion

The purpose of the investigation was to examine methods of nurse educators who were implementing the pedagogy of scenario-based case studies in their didactic undergraduate nursing courses. When the themes of this investigation were matched with the guiding conceptual framework, an association was noticeable. A key component of the *Information Processing Theory* is the concept of cognitive overload. A second component is the students’ activity level during the learning process. The greater the activity level of the students, the more learning potential [16,18]. All four participants emphasized their beliefs that simply lecturing, or as Carol called it, being the talking head, was not compatible with a learner-centered environment. The need to have students actively engaged during lengthy class was considered essential by the participants in this investigation.

The second conceptual framework that guided this research was developed by Marzano and is entitled *The Dimensions of Thinking Framework*. Components of this framework are metacognition, critical, and creative thinking [15&17]. According to the participants within this investigation, it was the transfer of responsibility for learning from teacher to learner that was evident when case studies were implemented. The transfer of responsibility is what encourages and enhances life-long learning for nursing students, thus allowing them to function adequately in the complex healthcare setting upon graduation. The reasons cited for this successful transfer were that student nurses learn to analyze systematically complex situations in a safe environment (classroom) and are able integrate the knowledge into their future practice as nurses with greater ease, which relates to the concepts of metacognition and critical thinking.

### Limitations

Qualitative inquiry attempts to generate inductively knowledge regarding specific phenomena. The real world, rather than the laboratory, is the setting for this type of research [21]. Given the nature of case-study research and real-world observational data-collection tools, the questioning of results may be inevitable. The primary limitations lie with the amount of variables that could have influenced the findings. One variable that could have influenced the results of this study was an inaccurate reporting of pedagogies by each participant during the interview process. Another potential limitation was that during the scheduled observation day, any participant could have inflated or exaggerated her performance during the lecture.

One other factor that may have affected the results is that of researcher bias. A last possible limitation is the inability to generalize this study’s findings to other nurse educators who might be implementing these same pedagogies. The small sample size (n = 4) is the primary reason for this.

## Discussion

The need for nurses to exhibit a higher standard of clinical competence is a direct result of increased technology, new treatment regimes, and complex co-morbidity disease processes of patients. Contributory disease processes and advanced technology in the acute-care setting have sounded an alarm among nurse leaders with respect to nursing education. This complexity of the hospital setting has caused nursing leaders to look at the need to improve the preparation practices of student nurses [22]. Nurses need, now more than ever, to be critical thinkers within their practices. Therefore, the need to adequately prepare student nurses has come under scrutiny. It has been stipulated that exposure in nursing school to the practice of analyzing, synthesizing, evaluating, and then responding quickly to patients’ needs should be a priority of nurse educators [22]. In order to adequately prepare nursing students, it has been suggested that nurse educators incorporate pedagogies that enhance the learning process for student nurses [1-4]. Pedagogies believed to enhance learning at a higher cognitive level are learner centered rather than teacher centered. Thus, pedagogies designed to enhance student nurses’ levels of thinking are those teaching methods that engage student nurses in the learning process. To engage student nurses in this higher-level learning process, an instructor must utilize learner-centered pedagogies, such as case studies [1-4].

The logical first step was to examine effective nurse educators in action. The explanation of how the participants within this study used the pedagogy of case studies will act as a conduit for other nurse educators attempting to implement learner-centered pedagogies. The themes that developed from this investigation did, indeed, shed light on the actual implementation strategies used for the pedagogy of case studies. The finding that the pedagogy of case studies was implemented each day informally via narrative pedagogy by each of the participants was instructive. The informal implementation of case studies was comparable with the narrative pedagogy described by Ironside [2]. Each of the participants was aware of the research supporting the use of these informal cases studies, which are analogues to personal experiences of the educators. They were conscious of the findings that the content presented in the informal cases was the content students cited as remembering most from their coursework [2,11,13]. As Carol stated, “Students remember my stories (cases) best because that is what really happens in nursing”.

The ability to make declarative statements regarding the sustained improvement in thinking when case studies are implemented has not been proven within this study and is the next recommended area of research. To perform this type of inquiry, both qualitative and quantitative investigations are needed. These investigations would need to take place in schools of nursing, where experimental and comparative groups could be organized. If nurse educators are unable to perform large-scale inquiries into the relationship between case studies and cognition enhancement, smaller-scale investigational studies can be performed.

## Competing interests

The author declares that he/she has no competing interest.

## Pre-publication history

The pre-publication history for this paper can be accessed here:

http://www.biomedcentral.com/1472-6955/12/15/prepub

## References

[B1] Del Bueno D A crisis in critical thinking Nurs Educ Perspect 2005 26 5 278 282 16295306

[B2] Ironside PM New pedagogies for teaching thinking: the lived experiences of students and teachers enacting narrative pedagogy J Nurs Educ 2003 42 11 509 515 14626389 10.3928/0148-4834-20031101-09

[B3] Ironside PM “Covering content” and teaching thinking: deconstructing the additive curriculum J Nurs Educ 2004 43 1 5 12 14748529 10.3928/01484834-20040101-02

[B4] Ironside PM Teaching thinking and reaching the limits of memorization: enacting pedagogies J Nurs Educ 2005 44 10 441 449 16268040 10.3928/01484834-20051001-02

[B5] Valiga TM Teaching thinking: is it worth the effort? J Nurs Educ 2003 42 11 479 481 14626382 10.3928/0148-4834-20031101-03

[B6] Walsh CM Seldomridge LA Critical thinking: Back to square two J Nurs Educ 2006 45 6 212 219 16780009 10.3928/01484834-20060601-05

[B7] Keating SB Curriculum development and evaluation in nursing 2006 New York: Lippincott Williams and Wilkins

[B8] Bastable S Nurse as educator: Principles of teaching and learning for nursing practice 2008 Boston: Jones and Bartlett Publishing

[B9] Staib S Teaching and measuring critical thinking J Nurs Educ 2003 42 11 499 508 10.3928/0148-4834-20031101-08 14626388

[B10] Tanner CA Thinking like a nurse: A research- based model of clinical judgment in nursing J Nurs Educ 2006 45 6 204 211 16780008 10.3928/01484834-20060601-04

[B11] Diekelmann N Narrative pedagogy: heideggerian hermeneutical analyses of the lived experiences of students, teachers, and clinicians Adv Nurs Sci 2001 23 53 71 10.1097/00012272-200103000-00006 11225050

[B12] Kern CS Bush KL McCleish JM Mind-mapping care plans: integrating an alternative to traditional care plans J Nurs Educ 2006 45 4 112 120 16629279 10.3928/01484834-20060401-04

[B13] Van Eerden K Using critical thinking vignettes to evaluate student learning Nurs Health Care Perspect 2002 22 10 231 234 15957399

[B14] Yoo MS Yoo IY The effectiveness of standardized patients as a teaching method for nursing fundamentals J Nurs Educ 2003 42 10 444 448 14577730 10.3928/0148-4834-20031001-06

[B15] Marzano RJ Designing a new taxonomy of educational objectives 2001 Thousand Oaks, CA: Corwin Press, INC.

[B16] Ormrod JE Educational psychology: Developing learners(4th ed.) 2003 Upper Saddle River: Merrill Prentice Hall

[B17] Marzano RJ Brandt RS Hughes CS Jones BF Presseisen BZ Rankin SC Suhor C Dimensions of thinking: A framework for curriculum and instruction 1988 Alexandria, VA: Association for Supervision and Curriculum Development

[B18] Shimamura AP What is metacognition? The brain knows Am J Psychol 2000 113 1 142 147 10.2307/1423465

[B19] Ausubel DP The psychology of meaningful verbal learning 1963 New York: Grune & Stratton

[B20] Bogdan R Biklen S Qualitative research for education; an introduction to theories and methods 2007 Boston: Pearson Education Inc.

[B21] Patton MQ Qualitative research & evaluation methods 2002 Thousand Oaks, CA: Sage Publications, Inc.

[B22] American Association of Colleges of Nursing The essentials of baccalaureate education: For professional nursing practice 2009 Washington, DC: Author

[B23] Benner P Sutphen M Learning across the profession: the clergy, a case in point J Nurs Educ 2007 46 3 103 108 17396548 10.3928/01484834-20070301-03

[B24] Kember D Gow L Orientations to teaching and their effect on the quality of student learning J High Educ 1994 65 1 58 74 10.2307/2943877

[B25] Kowalczyk RT Review of Teaching Methods & Critical Thinking Skills Radiological Technology 2011 83 2 120 132 22106387

